# Why do We Find It so Hard to Discuss Spirituality? A Qualitative Exploration of Attitudinal Barriers

**DOI:** 10.3390/jcm5090077

**Published:** 2016-09-01

**Authors:** Megan Best, Phyllis Butow, Ian Olver

**Affiliations:** 1 Psycho-Oncology Co-operative Research Group (PoCoG), University of Sydney, Sydney 2006, Australia; phyllis.butow@sydney.edu.au; 2 Division of Health Sciences, University of South Australia, Adelaide 5001, Australia; ian.olver@unisa.edu.au

**Keywords:** spirituality, religion, physician-patient relations, Neoplasms, communication barriers, qualitative research

## Abstract

**Background:** Despite known health benefits of spiritual care and high patient interest in discussing spirituality with their physicians, the frequency of spiritual discussions in the medical consultation is low. We investigated spiritual conversations for doctors caring for patients with advanced cancer; why these conversations so difficult; and what the underlying challenges are for discussing spirituality with patients; **Methods:** Participants were contacted through the Australian and New Zealand Society of Palliative Medicine and the Medical Oncology Group of Australia, including physicians from two secular countries. Semi-structured interviews were taped and transcribed verbatim. The text was analyzed using thematic analysis; **Results:** Thematic saturation was reached after 23 participants had been interviewed. The following themes were identified: (1) confusing spirituality with religion; (2) peer pressure; (3) personal spirituality; (4) institutional factors; (5) historical factors; **Conclusion:** This study explored the underlying attitudes contributing to the reluctance doctors have to discuss spirituality in the medical consultation. Underlying confusion regarding the differences between religion and spirituality, and the current suspicion with which religion is regarded in medicine needs to be addressed if discussion of spirituality in the medical consultation is to become routine. Historical opposition to a biopsychosocial-spiritual model of the human being is problematic.

## 1. Introduction

A paradox exists with regard to the discussion of spirituality by doctors and their patients, where spirituality refers to the way people engage with the purpose and meaning of human existence and shapes their personal values [1]. The literature reflects a high level of patient interest in discussing spirituality in the medical consultation, but a low frequency of doctors raise the topic [2,3]. This is despite several studies reporting that a majority of doctors believe that routine spiritual care (SC) would have a positive impact on patients, where SC refers to taking an interest in patient spirituality and providing support [3,4,5,6,7]. Spirituality should not be confused with religion, which is an organized form of spiritual expression and a subset of human spirituality as a whole.

Patients do not necessarily want their doctors to be spiritual advisors, but to enquire about patient spirituality in order to get to know the patient holistically, building the trust that allows them to ask difficult questions. Generally, referral to a chaplain is expected if spiritual problems exist [8]. This can be accommodated in those countries where end of life care is supported by multidisciplinary services. Benefits of spiritual discussions in the medical encounter include a strengthening of doctor-patient relationships [8], allowing the doctor to understand how patient beliefs influence decision-making [9] and to accommodate the patient’s religious beliefs in management [10,11]. Furthermore, spiritual support from the medical team is predictive of less aggressive care at the end of life (EOL) [12], an outcome not achieved by chaplaincy support alone.

Doctors’ discomfort and concerns regarding the appropriateness of SC provision in the face of high patient approval raises the possibility that some barriers are anticipated rather than experienced as problematic. What is it that causes so much uneasiness for doctors contemplating the discussion of spirituality with their patients?

A recent review found that the most commonly cited barriers to discussing spirituality and religion were lack of knowledge and/or training, lack of time, and personal discomfort of the physician [13]. Training in SC is associated with increased provision [14], and provides an avenue for improving SC in healthcare. With regard to time factors, Ellis and colleagues found that increased longevity of patient admission did not increase the frequency of spiritual discussion with inpatients [5], and Balboni and colleagues found that lack of time was not predictive of reduced frequency of SC provision by physicians. In the latter study, three barriers associated with less frequent SC provision were a perception that SC is not part of the professional role, that it was an inappropriate medical activity, as well as lack of training [15]. It was suggested by the authors that a key barrier to more frequent SC at the EOL has been a more basic conceptual question related to the relationship of medical practice and religion and spirituality, citing it an inappropriate medical role and risks to patient autonomy as arguments that have been used in support [16]. Other authors have noted the historical divide between science and religion in this context [17,18,19]. Interest in the impact of personal religiosity of medical practice of physicians has further highlighted the difficult relationship experienced between practice exclusively guided by the strictly scientific paradigm of evidence-based medicine and a personal faith-based religious adherence [20,21].

In undertaking this study, we sought to understand the challenges inherent to the spiritual discussion and explore the underlying reasons for the reluctance to embrace spirituality within healthcare, generally. In order to do this, we interviewed doctors caring for patients at the EOL, a time known to be associated with increased spiritual need, about the challenges of discussing spirituality in the medical consultation [22]. A previous report of this study outlined the process used by experienced practitioners in taking a spiritual history [23]. This paper focuses on an emerging theme we identified during the interviews, namely, doctors’ perceptions of stigma surrounding discussions of spirituality in the healthcare setting. We set out to explore whether this attitude towards spirituality existed in healthcare. It was not an a priori objective for the study.

## 2. Experimental Section

The empirical component of this project was a qualitative study using thematic analysis according to the method of Braun and Clarke, as described below [24]. According to this method, patterns or themes are identified and organized within the dataset in order to identify and examine the underlying ideas and assumptions informing the data. Analysis comes from a constructionist paradigm, where broader meanings are theorized as underpinning what is actually articulated by research participants.

### 2.1. Population

Eligible participants were doctors who cared for stage IV cancer patients (incurable disease) and practice in Australia or New Zealand.

### 2.2. Sampling Strategy

The researchers approached two professional groups, the Medical Oncology Group of Australia and the Australia and New Zealand Society of Palliative Medicine (ANZSPM), to advertise the study. Both groups are comprised of physicians who care for stage IV cancer patients. Potential participants were asked to contact the lead researcher by email to learn more about the study, ask questions, and be invited to participate. After informed consent was obtained, a time for a telephone interview was arranged.

All initial respondents were members of ANZSPM. Purposive sampling was used in order to guarantee a diversity of participants in terms of medical specialty, clinical experience, and interest in spirituality. By studying extreme (deviant) cases, researchers can often gain a better understanding of more regular patterns of behaviour, which allows exploration of each code until it is well understood. Deviant cases were identified by a technique known as snowballing, where existing study subjects are asked to help recruit future subjects from among their acquaintances, whose views are known to them. Two oncologists refused the invitation to participate, and reasons were not sought.

### 2.3. Data Collection

Where consent was obtained, one researcher (MB) conducted a 20–45 min semi-structured interview (see Table 1). Following the interview a demographic survey was completed. Interviews were digitally recorded and transcribed verbatim.

### 2.4. Analysis

Using line-by-line coding, the researchers developed initial codes to detail the processes involved in spirituality discussions in medical consultations. These initial codes were used to synthesize groups of data into focused codes which were applied to further transcripts. Using the constant comparative method, new codes were written as required and codes were collated into potential themes. Data collection and analysis occurred concurrently as themes were refined and applied to the data. In this way information was collected about stigma in SC after it was volunteered by several participants. Analysis continued until theoretical saturation was reached.

Rigor was derived from successive rounds of discussion and development of focused codes, definitions and themes and review of the coding process by all authors until theoretical coding was complete.

Ethics approval was given by the University of Sydney Human Research Ethics Committee (2014/156). Results were reported according to COREQ guidelines [25].

## 3. Results

Thematic saturation was reached when 23 participants were interviewed. Demographic details can be found in Table 2. There was an awareness among all the interviewees that attempts to discuss spirituality in medicine could provoke opposition. Discussion of the nature of this opposition generated data reported here. Five themes were identified: (1) confusing spirituality with religion; (2) peer pressure; (3) personal spirituality; (4) institutional factors; and (5) historical factors. 

### 3.1. Confusing Spirituality with Religion

Some participants described patients confusing spirituality with religion, and objecting to discussing spirituality due to a rejection of religion. This was also true for several participating doctors.

‘I think patients can get (spirituality) confused with religion, I think not…just how the question is posed, but…it depends very much on the patient’s own experience. So, when…we make a referral to pastoral care and try to explain what that is…some people, because of their own past experiences or their own belief…immediately relate to that as a religious thing. Now that isn’t necessarily conveyed by the staff but it can be a preconceived idea…For some patients…religion, if it’s not an attractive thing or they’ve had a past experience that’s difficult or they feel some guilt around it, then…there can be some blockage.’

—Male GP, 20 years’ experience (i.e., treating advanced cancer patients).

This may or may not be associated with a concern regarding proselytising.

‘I work for a Christian organization (and) some people find that confronting…if they think that people talking about religion are crossing a boundary…they feel vulnerable. And they’re worried that someone might be trying to convert them…On occasion I’ve had people say to me they don’t want to see the pastoral care worker…They might have had a lot of existential distress, but because they come from a strong atheist point of view, it’s a terrifying thing for them to be coming across anyone who has anything to do with religion.’

—Female palliative care specialist, 21 years’ experience.

### 3.2. Peer Pressure

Some doctors with faith mentioned that they had experienced disapproval from peers when they talked about their own faith with patients, or exhibited religious behaviour. This was described by some participants without faith as ‘uncomfortable’ behaviour.

‘In New Zealand…—it’s slightly more difficult…the staff would say, “We’re not really supposed to talk about our personal faith or anything like that here.” Whereas in UK, I was so open about that—if somebody asked you about it, you could talk about it.’

—Female palliative care specialist, 10 years’ experience.

‘I’ve never…been involved with doctors who’ve said “I’ll pray for you” because of their religious beliefs…So I don’t know if it’s inappropriate, I don’t know if that’s the right word, but I guess I would find that uncomfortable.’

—Female palliative care specialist with 13 years’ experience.

Discussion of the patient’s religion could be confused with discussion of the doctor’s own religion by hearers, so that any mention of religion was viewed negatively.

‘I think there’s some anxiety around it, I think people don’t want to be seen to be proselytising and I think people will associate religion with all sorts of structures that don’t fit in the health care sector. Yes, so I think there is a stigma.’

—Female palliative care specialist, 19 years’ experience.

However, the reaction to discussion of faith was also described as one of unfamiliarity as much as disapproval.

‘I think if you start talking about religion and faith in a tertiary hospital, a number of your colleagues will look sideways at you and think, “What’s that got to do with medicine?...But having said that, a couple of the grand rounds that I’ve done have involved a little bit of talking about religion. And it hasn’t been a bad thing. So it may be more theoretical than real. Maybe if we did talk about faith more, people would feel that it’s okay.’

—Male palliative care specialist, 36 years’ experience.

### 3.3. Personal Spirituality

Doctors of strong personal faith were conscious of the impact it could have on their practice of medicine. There was an awareness that their world view influenced the way they viewed spirituality, generally, and that they had to take care to be open to alternate views of spirituality in their patients.

‘I come from a Christian paradigm and…we can’t help but see things through our lens, but I have to be open to what other people experience as their spirituality’.

—Male palliative care specialist, 10 years’ experience.

At the same time, they thought that their faith allowed them to be more comfortable in discussing spirituality and issues like death and dying, particularly with patients who shared their faith.

‘Probably the most important thing is that I’m comfortable with my own beliefs and recognising that my beliefs don’t matter to the patient other than that I’m comfortable in them and, therefore, able to talk about death and dying in a way that allows them to support their beliefs, whatever they are, or help them to find a framework within which they can live out their life.’

—Female palliative care specialist, 6 years’ experience.

However, doctors of strong personal faith were also conscious of the potentially conflicting roles of evangelist versus their role as a professional. Some doctors expressed an existential dilemma when facing the need to encourage a patient in beliefs they believed to be misguided, or even harmful. However, regardless of their enthusiasm for their own faith, all were conscious of the need to avoid proselytizing without invitation.

‘I’ve got to admit when I first started out, I felt really uncomfortable because I didn’t want to impose…my beliefs on them. But now I have more confidence in opening up that discussion if they want to and just-well, teasing them with the concepts to see if they want a bite out of it because it’s really up to them to guide where we’re going. I want to talk about what’s important to them, but I also want to make sure that I’ve opened up areas that they may have felt a bit embarrassed to talk about that they really want to talk about.’

—Male oncologist, 33 years’ experience.

For some, the desire to avoid pushing their own beliefs onto patients could make them avoid discussing spirituality altogether. Others took opportunities as they arose on the initiative of the patient. Care was ultimately patient-centred and the needs of the patient were of priority in this cohort.

‘I think (proselytizing) is inappropriate…People have said to me ever since I started doing palliative care and I was a Christian, “Oh, what a wonderful mission field.” And I’ve always kind of gone, “Oh, I’m really uncomfortable about that.” So I see…patients are very vulnerable. So suddenly, if a patient brought up salvation and wanted me to pray for them and wanted to be a Christian, then I’d be very happy to oblige. But I feel very uncomfortable about manipulating people into a deathbed conversion. I view my role as to improve a patient’s experience of having cancer. Palliative medicine specialist; that’s fundamentally what I’m trying to do. And so it might include some attention to spiritual things in the broadest sense. And a lot of the time, I’m actually trying to honour their spiritual traditions. So…if I have a Catholic patient who’s dying, one of the things I would…automatically check for is evidence of the priest having visited. And if they haven’t, I’ll ask them do they want a priest to come. So if I go in there with a mission to proselytise, then I’m not able to do that. And I guess everybody…has to actually…sit and think about where they stand on that. So I’m employed by a secular organisation to provide secular assistance. And…while I’m happy to include spirituality in that because I think that’s part of the patient’s experience, I think it would be almost in breach of contract were I to be there with a primary mission of proselytising. So, I don’t do it.’

—Female palliative specialist, 30 years’ experience.

### 3.4. Institutional Factors

Discussion of spirituality was clearly more acceptable, even promoted, in faith-based institutions. Chaplains tended to be plentiful in such hospitals. One participant noted that the availability of a chaplain could alter the acceptability of overt religious behaviour, such as doctors praying with patient, even in a secular establishment, it being more tolerable when pastoral care services were absent.

‘So it’s a bit more that the chaplain has a job and that’s what they do and the doctor has a different job. Whereas in (my last job), I kind of combined both because our chaplain wasn’t as available.’

—Palliative care specialist, 10 years’ experience.

### 3.5. Historical Factors

The impact of the separation of science and spirituality on medical training since the Enlightenment was noted. This was described as resulting in an environment where the spiritual side of patients was largely ignored in the clinical setting.

‘I think even asking about it is taboo, because it’s not in our concept of healthcare...One of the problems in the medical model is this thing that’s always credited to Descartes, of the mind-body split and dualistic medical thinking, where we focus a lot on the body, and the mind is the realm of psychiatrists. And the connections between the mind and the body, Descartes didn’t know about, and it’s only very recently that our level of knowledge got to the point where we’re starting to understand mind-body connections. But actually, if you read Descartes’s original stuff, he actually talked about mind, body, and spirit. But the medical profession just conveniently forgot about spirit, I think, because it was all too hard. And certainly, today I don’t think we understand what spirit is, and I certainly don’t think we understand the connections between the mind and the body and the spirit in our understanding of a human being. And so I don’t know that we know how to diagnose a broken spirit. So I think the whole area of spirituality is taboo because it’s ignored, because it’s something we haven’t come to grips with. And I don’t think we know how to come to grips with it—it’s not something we can measure on a functional MRI…’

—Female palliative care specialist, 30 years’ experience.

The majority of participants were, nonetheless, conscious of the importance of spirituality for many of their patients and addressed it as they thought appropriate. The established practice of holistic care in EOL settings prevailed for this cohort.

‘My understanding, because I believe in holistic care, is that if I need to talk about sexuality, I should talk about sexuality. If I need to talk about faith, I should talk about faith. If I need to talk about bowels, I should talk about bowels, and I'm not going to bring those conversations into every consultation. Some people would say that in every initial consultation a question about people's sexuality…is standard. Well—that's not what I've done either. So, there's a few things where I wait for signals from patients before taking these things on as a really important conversation, but they're not necessarily things that I will talk through every time or with every patient.’

—Male oncologist, 23 years’ experience.

## 4. Discussion

In this qualitative study we examined the aspects of spiritual discussions that doctors find challenging. We found that participants were aware of several factors that could influence the acceptance of SC in the healthcare setting.

A prominent theme was that of confusion between religiosity and spirituality, with rejection of the former sometimes leading to inadvertent refusal of the latter. Dominant paradigms in society change over time, and currently individualism and personal autonomy are highly valued in Western societies. As a result, organized religions (as well as other authoritative institutions) are often regarded with suspicion. Possibly, inhumane acts performed in the name of religion on the current international stage also contribute to the negative way religion is portrayed in the mass media. This is unfortunate, as the condemnation of religion can obstruct what is really about helping patients find peace and meaning in their illness experience [26].

While spirituality has been noncontroversial as a component of palliative care provision, with the incorporation of palliative care into mainstream medicine, it has become more contentious [18,27]. This may be, in part, due to the increased availability of pastoral care in the palliative care setting making its provision less of a physician’s task there. The influence of context on spiritual care, such as the type of institution and availability of support services such as chaplains may imply that improvement of SC may require organizational, as well as attitudinal, change.

However, physician training needs to clarify the role of spirituality in patient care, and to clarify that discussion of spirituality does not have to involve proselytizing and/or violation of patient autonomy. Most doctors in this study did not have this conflict. The sharing of scripture or life-stories on the part of the doctor is not patient-directed and is a boundary violation within the ethics of care. It was recognized by this cohort that it is inappropriate for doctors to take advantage of the relationship imbalance between doctor and patient to say things that offended the patient or constituted proselytizing. Guidelines to help avoid such behaviour are available [28,29,30], and will benefit those with and without personal faith.

It also needs to be recognized that medicine is a moral practice and that doctors’ worldviews, whatever they are, will inevitably affect their ethics and their clinical practice [20]. Non-religious doctors may not be part of an organized belief system, but that does not mean they are morally neutral. Everyone is spiritual (whether they like it or not) as even if they avoid religion, they cannot escape the questions about the meaning of existence that come to us all at some time. Spirituality must be addressed for those who need to find peace at a time of existential distress. This is often what the doctor would aim to achieve for the patient and would seek for him/herself in a similar situation. Spiritual needs have been identified in patients who did not consider themselves ‘spiritual’ [31]. A doctor’s own faith, therefore, need not necessarily cause undue concern.

The historical separation of spirituality and science makes discussion of spirituality in healthcare unexpected (as a result of medical training which largely excludes it [32,33,34]), but most participants in this study did not experience actual opposition. Given that both science and spirituality both influence health [35], it is not valid to practice one to the exclusion of the other. 

Introduction of a biopsychosocial-spiritual model of care [36] has been justified within the empirical requirements of science and is required for holistic patient care. Indeed, spirituality is increasingly taught in medical schools but not (yet?) translating to care in clinical settings. This study suggests that an attitudinal change is required before spirituality is routinely included in patient history-taking. The final quote of the study identified a helpful parallel. Human sexuality is very important to patients, even during sickness, but discussion causes discomfort for doctors and is not routinely included in medical consultations. Ditto spirituality. However, there will be occasions when to ignore the spiritual dimension is to ignore one of the most important factors contributing to patient distress, or one of the most important coping mechanisms available to them [8,23]. While comprehensive SC may not be a skill of many practitioners, the ability to screen for spiritual problems should be a minimal requirement of doctors. Greater discussion of spiritual needs of patients should help normalize these conversations and reduce any perception of stigma.

Qualitative research is not generalizable but intends to identify processes for further study. This analysis included a small sample of physicians and other healthcare providers may give different responses.

## 5. Conclusions

This study explored the underlying reasons for the reluctance doctors have to discuss spirituality in the medical consultation. As well as providing the necessary communication skills to physicians, underlying confusion regarding the differences between religion and spirituality, and the current suspicion with which religion is regarded in medicine needs to be addressed if discussion of spirituality in the medical consultation is to become routine.

## Figures and Tables

**Table 1:** INTERVIEW SCHEDULE *.

1	Spirituality is an important domain in the quality of life of many cancer patients. What do you think might be meant by the term spirituality in relation to your work with cancer patients?
2	Tell me about your views on discussing spirituality with patients who have metastatic disease.
3	If you wanted to discuss spiritual needs with a patient, how would you go about doing it? What would help you?
4	What would make it difficult?
5	Imagine you could give your younger self tips as you start out in medical practice about discussing these issues with your patients. What advice would you give yourself?
6	We’re just about finished—is there anything else you would like to add?

* These are the initial questions used in semi-structured interviews which were adapted as required to develop themes identified during the study analysis. After the theme of stigma was identified, participants were asked directly whether they thought there was stigma associated with spiritual discussions in healthcare.

**Table 2:** Demographic characteristics of sample.

Characteristic	Total *n* = 23 (%)
Mean age (years)	55.2
Mean years’ experience	21.5
Female	8 (34.8)
Previous formal training in SC	7 (30.4)
**Specialty**	
Palliative Medicine	15 (65.2)
Oncology	5 (21.7)
General Practice	3 (13.0)
**Country of Birth**	
Australia	14 (60.9)
Europe	7 (30.4)
New Zealand	2 (9.7)
**Religious Affiliation**	
Christian	14 (60.9)
Nil	9 (39.1)
**Self-Reported Religiosity and Spirituality**	
How important is religion to you?	
Not at all	8 (34.8)
Moderately	8 (34.8)
Very	7 (30.4)
How important is spirituality to you	
Not at all	0
Moderately	5 (21.7)
Very	18 (78.3)
